# Editorial: March 2022: Ovarian and prostate cancer awareness month

**DOI:** 10.3389/fendo.2023.1133963

**Published:** 2023-02-13

**Authors:** Marzia Di Donato

**Affiliations:** Department of Precision Medicine, University of Campania “L.Vanvitelli”, Naples, Italy

**Keywords:** prostate cancer, ovarian cancer, prolactin, gynaecological cancer, ion channel related genes, SARS- CoV2

Prostate cancer (PC) and ovarian cancer (OC) are two hormone-related types of cancer with high incidence. PC is the fifth most common cause of cancer-related mortality among men and the second most common type of cancer worldwide, while OC ranks fifth in cancer deaths among women. In both cases, early detection and diagnosis allow for more treatment options, increasing the chances of survival. Thus, routine checkups are encouraged for successful prevention.

These types of cancers are united by a lack of symptoms in their early stages. The key to early detection is the persistence of symptoms. The main symptoms of OC are pelvic pain, bloating and increased stomach size, feeling full quickly or difficulty eating, and urinating more frequently. The typical symptoms of PC are trouble with urination or frequent urination, blood in the urine or semen, and pain in the spine/hips/ribs. Unfortunately, no specific screening tests for early detection are available in either case, so new markers must be sought, and new challenges must be faced.

This special issue presents a collection of five papers: one review article and four original research articles pertaining to PC or OC.

Ramírez-de-Arellano et al. focus their interesting review on the role of prolactin (PRL), a hormone produced by the pituitary gland and multiple non-pituitary sites, and its implication in oncogenic processes. In particular, they describe the PRL/PRLR axis in cancers of the female reproductive system, such as cervical, ovarian, and endometrial cancers, highlighting its central involvement and significance and the need for further investigation.

It is recognized that there are multiple subtypes of OC, depending on the cell types that are involved: epithelial, germ cell, and stromal. Among these, epithelial ovarian cancer (EOC) is the most common type of OC, and this category also covers fallopian tube cancer and primary peritoneal cancer. This type of cancer is the most lethal cancer among gynecological malignancies, and it is classified into two main groups, Types I (low-grade) and II (high-grade), based on their distinctive clinicopathological and molecular features (1). In this context, Achlaug et al. analyze the expression of ZYG11A, initially identified as a cell cycle regulator, in a panel of EOC specimens. They demonstrate that high ZYG11A levels correlate with low EOC histological grade and, conversely, low ZYG11A levels are associated with high-grade EOC, thus identifying the ZYG11A gene as a putative tumor suppressor.

Connecting their work to the highly topical subject of severe acute respiratory syndrome coronavirus 2 (SARS-CoV-2), Li e al. present an article analyzing the negative impact of severe forms of SARS-CoV-2 in patients already affected by uterine corpus endometrial carcinoma (UCEC). Patients affected by cancer may experience immunological suppression (2), and SARS-CoV-2 is responsible for an increase in the death toll. Forms of UCEC, characterized by malignant metastasis, are increasing around the world, and current therapies used in the treatment of UCEC patients are less effective when over-infection with SARS-CoV-2 occurs, causing an undesirable increase in mortality. Through bioinformatic and computational approaches and molecular docking data, the authors analyze the role of PLB, a naturally occurring naphthoquinone, as a possible drug for use in the potential treatment of UCEC patients infected with SARS-CoV-2 in the current evolving situation. The features that they identify are likely to be associated with the anti-inflammatory effects exerted by PLB, given the systemic inflammation that leads to multi-organ failure in SARS-CoV-2 (3).

Currently, several therapeutic approaches are available for the care of PC patients. Nevertheless, PC may evade these treatments and often spreads, with a significant increase in mortality rate. Gao et al. analyze, through a large-scale study of patients undergoing health checkups in China, the intersection between PC and metabolic syndrome (MetS), a metabolic disorder including hypertension, hyperglycemia, obesity, elevated triglyceride level, and decreased high-density lipoprotein cholesterol (HDL-C). The authors conclude that there is no significant correlation between MetS and PC, while older age and elevated PSA levels could be considered risk factors for PC.

In the context of PC, Luo et al. present the construction and validation of a novel signature based on genes related to voltage-gated chloride ion channels (CLCs) as prognostic biomarkers in PC management. The relevance of these genes is not unexpected, since voltage-gated channels, including calcium, sodium, chloride, and potassium channels, are involved in various metabolic and cellular processes. Thus, it is not surprising that they are implicated in numerous diseases, including cancer. The calcium channels, for example, mediate activation of several intracellular pathways by modulating the influx of cations. Some of these have recently emerged as important actors in PC pathogenesis. While TRPM3, TRPM4, and TRPM6 have been linked to non-transcriptional action of steroid hormones in various cell types, TRPM8 is directly related to non-genomic actions of androgens in PC progression (4, 5). In their paper, the authors observe through enrichment analysis that CLCN2 and CLCN6 are related to cellular and metabolic processes and could function as excellent independent prognostic factors. In addition, through the use of cellular models, they observe that downregulation of CLCN2 or CLCN6 suppresses mitochondrial function, regulating cell proliferation, invasion, and apoptosis by decreasing ATP production. Although the underlying molecular mechanisms remain unknown, the authors propose potentially powerful targets for the improvement of PC diagnosis strategies.

A plethora of steps forward have been taken in this field, and still more are needed. The analysis of new molecular mechanisms and identification of new diagnostic and prognostic markers is the major challenge that must be undertaken in order to enable the practice of a new precision medicine that takes into account individual differences in patients’ genes, environments, and lifestyles.

Given the incidence of PC and OC and the relative mortality associated with them, particularly when they progress toward malignant stages of the disease and spread, Frontiers has proposed this article collection to coincide with March, the corresponding Awareness Month. This proposal is important, not only because it offers the opportunity to disseminate knowledge about these types of cancer, but also because underlines the importance of researching innovative tools for early diagnosis and new treatment options.

## Author contributions

The author confirms being the sole contributor of this work and has approved it for publication.
